# Trust in automation and the accuracy of human–algorithm teams performing one-to-one face matching tasks

**DOI:** 10.1186/s41235-024-00564-8

**Published:** 2024-06-21

**Authors:** Daniel J. Carragher, Daniel Sturman, Peter J. B. Hancock

**Affiliations:** 1 School of Psychology, Faculty of Health and Medical Sciences, University of Adelaide, Adelaide, SA 5005 Australia; 2 Psychology, Faculty of Natural Sciences, University of Stirling, Stirling, Scotland, UK

**Keywords:** Identity verification, Human–computer interaction, Face recognition, Human factors, Collaborative decision-making

## Abstract

The human face is commonly used for identity verification. While this task was once exclusively performed by humans, technological advancements have seen automated facial recognition systems (AFRS) integrated into many identification scenarios. Although many state-of-the-art AFRS are exceptionally accurate, they often require human oversight or involvement, such that a human operator actions the final decision. Previously, we have shown that on average, humans assisted by a simulated AFRS (sAFRS) failed to reach the level of accuracy achieved by the same sAFRS alone, due to overturning the system’s correct decisions and/or failing to correct sAFRS errors. The aim of the current study was to investigate whether participants’ trust in automation was related to their performance on a one-to-one face matching task when assisted by a sAFRS. Participants (*n* = 160) completed a standard face matching task in two phases: an unassisted baseline phase, and an assisted phase where they were shown the identification decision (95% accurate) made by a sAFRS prior to submitting their own decision. While most participants improved with sAFRS assistance, those with greater relative trust in automation achieved larger gains in performance. However, the average aided performance of participants still failed to reach that of the sAFRS alone, regardless of trust status. Nonetheless, further analysis revealed a small sample of participants who achieved 100% accuracy when aided by the sAFRS. Our results speak to the importance of considering individual differences when selecting employees for roles requiring human–algorithm interaction, including identity verification tasks that incorporate facial recognition technologies.

## Significance statement

Automated facial recognition systems (AFRS) are computer algorithms that can compare the appearance of two faces to indicate whether they likely show the same person or two different people. The exceptional accuracy of many modern AFRS (often > 99.9%) has led to their integration into many identity verification scenarios, such as passport control. However, AFRS still make errors, meaning human involvement is often required. While we tend to be very good at matching familiar faces, the average human errs on 10–30% of trials in standard unfamiliar face matching tasks. As such, human involvement in AFRS decision-making is not certain to produce perfect task accuracy. We have previously shown that human–AFRS teams were outperformed by the same simulated AFRS alone, since humans often overturned correct decisions from the system, while also failing to detect or correct actual errors. In the current study, we investigated whether certain characteristics might make some participants better at this task than others. Specifically, we examined whether participants’ trust in automation was related to the level of face matching performance they achieved when assisted by a simulated AFRS. As expected, participants with greater trust in automation, or generally favourable attitudes towards the AFRS, achieved larger gains in performance when using the simulated AFRS as a decision aid, compared to participants with lower automation trust or greater self-confidence. Our results may have implications for the selection and training of professionals working in roles that require the evaluation or oversight of identity verification decisions made by facial recognition technologies.

## Introduction

There are many scenarios in which the human face is used for identification. For example, when travelling internationally, border control officers must decide whether a passport image matches the person presenting it for inspection. This is an example of a one-to-one face matching task, in which an observer must compare two faces—whether in the form of images, video, or live appearance—to determine whether they show the same person or two different people. These types of matching tasks can also occur in other contexts, such as buying age restricted products, opening bank accounts, or during interactions with law enforcement officers. Despite the prevalence of face matching tasks for identification purposes, decades of research have shown that the performance of the average human on this task is error prone when the faces are unfamiliar to the observer (Bruce et al., 1999; Burton et al., 2010; Kemp et al., 1997; Megreya & Burton, 2006).

Although there are substantial differences in unfamiliar face matching ability between individuals, the average observer commonly makes errors in 10–30% of judgments, depending on the specific face matching test used (Burton et al., 2010; Fysh & Bindemann, 2018b; White et al., 2022). However, the individual differences are such that some observers consistently achieve perfect, or near perfect, performance, while others barely surpass chance (Bobak et al., 2016b; Burton et al., 2010; White et al., 2017). Curiously, face matching ability is rather resistant to improvement via training (Towler et al., 2019; Towler et al., 2014; c.f. Towler et al., 2021), and employment in a professional role that requires making face matching decisions regularly is not certain to lead to improved performance (e.g. White et al., 2014; c.f. Wirth & Carbon, 2017). In addition to being somewhat error prone, the performance of human observers deteriorates as time on task increases (Alenezi et al., 2015), and when subject to time pressures (Fysh & Bindemann, 2017). These performance characteristics can be problematic in real-world contexts that require identity screening over prolonged periods of time, such as at airports.

Automated facial recognition systems (AFRS) are computer algorithms capable of performing identification tasks (i.e. comparing one image to an entire database), or verification tasks (i.e. comparing one image to another specified image). Here we are interested in their performance on verification tasks. While a detailed explanation of their computational structure is presented elsewhere (Noyes & Hill, 2021), we can summarise that these algorithms must locate a face in the submitted image, before processing the face to produce a vector of numbers that, in effect, describes the characteristics of the face. The algorithm then compares this vector to that created for the other submitted image. The comparison of these vectors generates a similarity value, which is evaluated against a threshold that has been calibrated to an acceptable level of false positive decisions (FRONTEX, 2015). Values to one side of the threshold are indicative of an identity match, while values to the other side signal an identity mismatch.

While the performance of early algorithms was comparable to average novice humans only on high-quality imagery (O'Toole et al., 2007b), many state-of-the-art algorithms (deep convolutional neural networks: DCNNs) are now comparable to—or surpass—the best human observers (Hancock et al., 2020; Phillips et al., 2018). While performance varies substantially between algorithms, and can be influenced by a variety of image factors, publicly available testing data shows that many of the top algorithms achieve accuracy > 99.9% on standardised databases of high-quality images (National Institute of Standards and Technology, 2024). Despite their impressive performance, these algorithms still make mistakes (Grother et al., 2021; Hancock et al., 2020). As such, a human is often required to be included in the decision-making process with AFRS (Fysh & Bindemann, 2018a; MacLeod & McLindin, 2011), a model of “human-in-the-loop” oversight that is a form of human–algorithm teaming (Howard et al., 2020).

Previous research has suggested that significant performance gains can be made by combining the independent identification decisions made by humans and algorithms (O'Toole et al., 2007a; Phillips et al., 2018). However, the few studies that have investigated the performance of interacting human–algorithm teams have not found such impressive performance gains (Barragan et al., 2022; Carragher & Hancock, 2023; Fysh & Bindemann, 2018a; Howard et al., 2020). Rather, these studies point to a pattern of sub-optimal use of the AFRS as a decision aid by the human operator (Bartlett et al., 2023). That is, they either disregard correct decisions from the system, or fail to correct errors from the system. Howard et al. (2020) reported that humans were biased to shift their identification decision towards that of the AFRS, regardless of the accuracy of the algorithm’s decision. This tendency was exacerbated when the faces were shown wearing face masks (Barragan et al., 2022). Similarly, Fysh and Bindemann (2018a) showed that human accuracy was higher on trials that were answered correctly by the AFRS, but lower on those that the algorithm erred on. The findings from these studies suggest that humans tend to follow the decisions from the algorithm, regardless of whether the decision is correct. We note here that these laboratory-based studies differ in their use of AFRS; Carragher and Hancock (2023) showed participants decisions from a “simulated” AFRS that were informed by the performance of a real algorithm on the same matching task, whereas both Fysh and Bindemann (2018a) and Howard et al. (2020) showed participants decisions from “AFRS” that was entirely fictitious (for further discussion, see Carragher & Hancock, 2023).

We have previously investigated changes in human face matching performance when assisted by a simulated AFRS (Carragher & Hancock, 2023). We use the term “simulated” AFRS to accurately convey that while a real DCNN was used to inform the performance of the simulated AFRS that was shown to participants in our experiments, we also introduced errors that the real system did not make, so that we could study participants’ ability to detect and overturn incorrect decisions from an algorithm. We use the abbreviation sAFRS from here on in to refer to the simulated AFRS shown to participants in our experiments. Across five experiments, Carragher and Hancock (2023) found that participants significantly improved their own face matching performance when shown the identification decisions from a sAFRS that was given accuracy above 90.5%. However, the performance of the human–sAFRS team was consistently sub-optimal, failing to reach the level of performance that the sAFRS achieved alone. Human operators tended to overturn correct decisions from the sAFRS, while also failing to correct the errors made by the decision aid. This consistent pattern of results demonstrated that, at least in this simplified model of human–sAFRS teaming, the human operator is a factor limiting the performance of the system (Carragher & Hancock, 2023; Heyer et al., 2018; White et al., 2015a).

To date, human–algorithm teaming research in the context of face matching has largely focused on addressing basic questions of collaborative performance when humans use AFRS as decision aids (Carragher & Hancock, 2023; Fysh & Bindemann, 2018a; Howard et al., 2020). These studies have not investigated whether there are individual differences in the effective use of these decision aids. Yet, factors that influence the use of automated decision aids more generally have been studied for decades in the field of human factors research (Lee & Moray, 1994; Parasuraman & Riley, 1997; Riley, 2018; Wickens et al., 2015). Among the many factors shown to influence automation use, including self-confidence (Riley, 1989) and workload (Parasuraman et al., 1993), trust in the automated decision aid is crucial to use of automated systems (Hoff & Bashir, 2015; Lee & See, 2004).

Trust in automation requires an appropriate level of calibration between the expectations of the operator and the capabilities of the automation (Lee & See, 2004). Excessive trust in an automated system can lead to overreliance and complacency (Wickens et al., 2015). That is, due to high levels of trust, operators might defer decision-making to the automated system, and subsequently fail to notice and correct errors made by the system (Dixon et al., 2007). Conversely, mistrust in the automated system can result in under reliance or disuse, whereby operators reject correct decisions from the aid (Parasuraman & Riley, 1997; Wickens, 1995). Consequently, in environments where there tends to be under reliance or disuse of a reliable automated decision aid, higher levels of trust in automation may be associated with better collaborative team performance.

Trust in automation is a multifaceted construct (Hoff & Bashir, 2015). While trust is dependent on the accuracy of the automation (Riley, 2018), trust can also be learned dynamically through experiencing the system’s accuracy (Ross et al., 2008). Perceptions of accuracy may also depend on the types of errors made by the automation. For instance, a system that makes errors that are obvious to a human might lead to a general distrust in the accuracy of its other decisions (Madhavan et al., 2006). Further, the extent to which trust in automation influences reliance on the system is influenced by the human’s confidence in their own ability to complete the task (Riley, 2018). If trust in the automation exceeds self-confidence, there is increased likelihood of reliance on the automated decision aid (Lee & See, 2004). These findings suggest that reliance on an AFRS may be dependent on the interplay between the human’s trust in the system and self-confidence in their ability to match faces.

The aim of the current project is to investigate how operators’ trust in automation might influence their use of a sAFRS in a one-to-one face matching task. From the literature reviewed above, our overarching prediction is that there will be a positive relationship between trust in the sAFRS and collaborative performance gains, such that participants who express higher levels of trust in automation will benefit most when using the sAFRS as a decision aid.

## Experiment 1

We begin by briefly reporting the results of new exploratory analyses of previously unreported data from Carragher and Hancock (2023), for the purpose of informing the pre-registered predictions that we make in Experiment 2. At the end of several experiments reported in Carragher and Hancock (2023), we asked participants exploratory questions about their beliefs regarding the accuracy of humans and algorithms on face matching tasks. Participants were also asked whether they thought that they were more accurate than an AFRS at face matching, and whether they would prefer to work with a human or an AFRS if they were to do the task again. It was beyond the scope of our first paper to examine these responses. Here, we analyse these data for the first time to investigate whether the responses to these questions were related to the level of collaborative performance participants achieved when assisted by the sAFRS.

## Methods

These analyses were conducted on the data of 101 participants (*M*_age_ = 32.1, SD = 11.0), combined from the AFRS conditions of Experiment 1a and Experiment 1b of Carragher and Hancock (2023). Briefly, these participants completed the Expertise in Facial Comparison Test (EFCT; White et al., 2015b) in two phases: an unassisted baseline phase, and an aided task phase where they were shown the identification decision made by an sAFRS prior to making their own response. The EFCT was divided into Set A and Set B (which each consisted of 42 match trials and 42 mismatch trials; see White et al., 2015b), the presentation of which were counterbalanced across the baseline and aided task phases between participants. The sAFRS was given an accuracy of 97.6% on the EFCT, such that it made 2 errors (1 match trial, 1 mismatch trial), in the aided task phase (82/84 correct). At the conclusion of the face matching task, participants were asked exploratory questions including “*Do you think that your face matching abilities are better than those of a computer system?*” (Definitely Not, Probably Not, Probably Yes, Definitely Yes; converted to binary responses of “no” and “yes” for analysis here), “*Imagine that you were going to do this task again. If you could choose your source of help, would you prefer to see the decisions made by a computer system or those made by another person?*” (computer, person), and to estimate the accuracy of themselves, the average human, the simulated facial recognition system from the experiment, and “the best facial recognition system in the world”, on a face matching task (0–100%). For further methodological details, please see Carragher and Hancock (2023). The measure of performance reported below is overall accuracy.

### Ethics

The original research (Carragher & Hancock, 2023) was conducted with the approval of the General University Ethics Panel at the University of Stirling. All participants gave their informed consent prior to data collection. The current line of research received ethical approval from the Human Research Ethics Subcommittee in the School of Psychology at the University of Adelaide.

## Results

While the average performance of all participants improved from baseline (*M* = 75.79, SD = 7.32) to the test phase (*M* = 84.97, SD = 9.31) with sAFRS assistance, (*t*(100) = 11.29, 95% CI [7.57, 10.80], *p* < 0.001, *d* = 1.12), participants who later reported that they would rather complete the face matching task again with the assistance of a sAFRS (*n* = 50) showed greater absolute change in overall accuracy (*M*_change_ = 12.86, *SD* = 6.90) than participants (*n* = 51) who had a preference for a human partner (*M*_change_ = 5.58, SD = 7.76), (*t*(99) = 4.98, 95% CI [4.38, 10.18], *p* < 0.001, *d* = 0.99). Similarly, participants who reported that the sAFRS was likely better at face matching than they were (*n* = 72) showed greater absolute change in performance when aided (*M*_change_ = 10.23, *SD* = 7.99) than individuals who thought they were more accurate (*n* = 29) than the sAFRS (*M*_change_ = 6.57, SD = 8.16), (*t*(99) = 2.07, 95% CI [0.16, 7.18], *p* = 0.041, *d* = 0.46). Finally, we created a relative estimated accuracy score by subtracting participants’ estimates of their own face matching accuracy (made as a percentage from 0–100%) from their estimate of the accuracy of the sAFRS in the experiment, such that positive values meant that the sAFRS was estimated to be more accurate. A significant positive correlation, *r*(99) = 0.23, *p* = 0.021, indicated that participants who estimated that the sAFRS was more accurate at face matching than they were showed greater improvement when using it as a decision aid.

## Discussion

The results of these exploratory analyses suggest that participants who held favourable beliefs about the capabilities of the sAFRS achieved larger performance gains when given the opportunity to use it as a decision aid. This pattern of results is consistent with previous research showing that automation use is greatest in situations where trust in automation is higher than self-confidence (Hoff & Bashir, 2015; Lee & Moray, 1994; Lee & See, 2004). However, we note here that none of these results are specifically about trust in automation. Rather, we are inferring that participants’ favourable responses towards the sAFRS are indicative of greater trust in automation. A second limitation to this experiment is that these exploratory questions were only asked at the end of the task, when responses may have been influenced by the experience of completing the face matching task or using the sAFRS (Madhavan et al., 2006; Ross et al., 2008). We address both limitations in Experiment 2.

## Experiment 2

We aimed to replicate and extend the exploratory results of Experiment 1 in a pre-registered experiment that was specifically designed to measure trust in automation. In Experiment 2, we used an expanded set of questionnaire items to measure different aspects of trust in automation, which were completed both before and after participants used the sAFRS. Responses to two questions regarding self-confidence and trust in automation allowed us to calculate Lee and Moray’s (1994) *relative trust in automation* measure, which is central to Hypothesis 4. We also asked participants explicitly whether they trusted the AFRS to help them with the task (binary response: yes, no). Finally, we sought to replicate the three exploratory results reported above in Experiment 1 using the same questionnaire items. All seven hypotheses below were pre-registered prior to data collection (see Data Availability). They can be separated into those (H1) that replicate Carragher and Hancock (2023), those that replicate the results of Experiment 1 (H5, H6, H7), and those that extend the results of Experiment 1 (H2, H3, H4).

### H1

Participants will improve their face matching performance compared to baseline when using the sAFRS as a decision aid. Despite this improvement, performance in the aided task phase will fail to reach the level achieved by the sAFRS alone (overall accuracy = 95.0%).

### H2

There will be a significant interaction between Task Phase (baseline, aided) and Trust (yes, no), such that the increase in face matching performance when aided will be greater for participants who trust the sAFRS than those who do not.

### H3

Despite the predicted effect of trust (H2), we also expect that the aided performance of both trust conditions (yes, no) will fail to reach the level of performance achieved by the sAFRS alone.

### H4

The change in performance when aided (as a percentage of improvement possible from baseline) will be positively correlated with relative trust in automation (Lee & Moray, 1994), such that greater trust in automation will be associated with greater improvement when using the sAFRS as a decision aid.

### H5

The change in performance when aided (as a percentage of improvement possible from baseline) will be positively correlated with relative estimated accuracy (a difference score calculated from participants’ estimates of their own accuracy and that of the sAFRS), such that positive values (the sAFRS is estimated to be more accurate than the self) will be associated with greater improvement when using the sAFRS as a decision aid.

### H6

Participants who indicate that the sAFRS is better at face matching than they are (when asked directly) will experience a greater increase in performance (as a percentage of improvement possible) when using the system as a decision aid than participants who believe that they are more accurate.

### H7

Participants who would rather complete the task again with an sAFRS partner will experience a greater increase in performance (as a percentage of improvement possible) when using the system as a decision aid than participants who indicate a preference for a human partner.

## Methods

### Sample size

Our pre-registration describes re-analysing the “preferred partner” result in Experiment 1 according to the revised analysis strategy outlined for Experiment 2 (H7). This result returned a Cohen’s *d* effect size of 0.78. An a priori power analysis (G*Power: Faul et al., 2007) indicated that 54 participants (total) were required to achieve 80% power to detect an effect of *d* = 0.78 in an independent samples *t* test. However, since trust in automation is an individual attribute that we expect to vary among our participants, we cannot randomly allocate participants to a particular trust condition. As such, we aimed to recruit a much larger sample of 170 participants, expecting that data from approximately 160 participants would be available for analysis once our exclusion criteria had been applied. This sample size would exceed those previously collected by Carragher and Hancock (2023), who had approximately 40–50 participants in each between-participants condition.

### Participants

All participants were recruited from the online research platform *Prolific* (https://www.prolific.com/). We received consent from 174 unique participants, who were all USA nationals living in the USA. As per our pre-registration, data were excluded from participants who: did not complete the face matching task (*n* = 7), failed an attention check face matching trial (*n* = 1), failed an attention check question (*n* = 3), attempted the face matching task more than once (*n* = 2), or who took too long to complete the task (*n* = 1). The final analysis included data from 160 participants (*M*_age_ = 37.6, *SD* = 12.2). The experiment took an average of 16:03 min to complete (SD = 7:12). Participants received £2.00 (approx. $2.41USD) for their participation, which is above the minimum payment rate of £6.00 ($8.00USD) per hour required by Prolific. Ethical approval was granted by the Human Research Ethics Subcommittee in the School of Psychology at the University of Adelaide.

### Glasgow face matching test 2

Participants in Experiment 2 completed the short version of the Glasgow Face Matching Test 2 (GFMT2-S; White et al., 2022). The GFMT2-S consists of 80 pairs of unfamiliar faces, which we split evenly into two sets (A, B) of equal difficulty (White et al., 2022). Each set consisted of 20 identity match pairs and 20 mismatch pairs. The presentation order (baseline, aided) of each set was counterbalanced between participants, such that half completed Set A at baseline, while the other half received sAFRS assistance on Set A. Within each set, trial order was randomised.

The two faces in each pair were presented simultaneously. During the baseline task, participants responded to the question “*Do these photographs show the same person, or two different people?*” with a 2AFC response (“same” or “different”). The trial display for the aided half of the task was nearly identical to that shown at baseline. The only change was that below the trial question, participants saw a new line that read “*Facial Recognition System Says:*”. Like the participants, the simulated AFRS gave identification decisions of “*SAME*” or “*DIFFERENT*”. Participants were then asked to give their own “same” or “different” response to each pair (as they did at baseline). The two faces remained onscreen until a response was given.

### Automated facial recognition system

#### Real DCNN

The decisions presented to participants from the sAFRS in the experiment were based on the performance of a real DCNN (that used in Carragher & Hancock, 2023) on the GFMT2-S (White et al., 2022). The real DCNN correctly resolved 78/80 trials in the GFMT2-S, making 1 error on a match trial, and 1 error on a mismatch trial. Please note that “match” and “mismatch” trials are also known as “mated” and “non-mated” trials, respectively, in the computer science literature. We continue to use the terms “match” and “mismatch” here, which are more common in the study of human face matching ability.

#### Simulated AFRS

To ensure that the sAFRS made an error on one match trial and one mismatch trial in each counterbalance condition of the GFMT2-S (Set A, Set B), we selected two additional pairs (one match, one mismatch) that the sAFRS would be seen to err on in the experiment. The pairs selected to be additional errors for the sAFRS were the trials that the real DCNN resolved correctly but received similarity values closest to the system’s decision threshold (i.e. those that were closest to being errors). The sAFRS erred on the same 2 pairs for all participants in each counterbalance condition (4 trials across the whole GFMT2-S), giving it an overall accuracy of 95% in this experiment. Participants were told the accuracy of the sAFRS in the task instructions (Carragher & Hancock, 2023). As the average accuracy of human observers on the GFMT2-S is 76.4% (SD = 10.0; White et al., 2022), we anticipated that an sAFRS with 95% accuracy would benefit most participants.

### Attention checks

#### Face matching trials

As in our previous work, we added an attention check trial to each set of the GFMT2-S (Carragher & Hancock, 2023; Carragher et al., 2022). These attention check trials were mismatched pairs of famous faces that could be correctly resolved by differences in gender or ethnicity. Data from participants who failed to correctly respond “different” to both attention check trials were discarded from all analyses. The sAFRS did not make an identification decision for the attention check trial in the aided task phase—rather, the system reported that it was “OFFLINE”.

#### Attention check question

At the conclusion of the experiment, participants were asked a multiple-choice question about the stated accuracy of the sAFRS in the task instructions (95% or 55%). All data were excluded from participants who responded incorrectly.

### AFRS example trials

New to the current study, participants were shown three example displays with decisions from the sAFRS before starting the aided task phase. The text below the example displays told the participants how to interpret the decision from the sAFRS and stated whether the sAFRS was correct on that example trial (the only time feedback was provided in the experiment). Participants saw the sAFRS give two correct answers (one match trial, and one mismatch trial), and one error (on a mismatch trial). These example pairs were unfamiliar faces from the Stirling Famous Face Matching Task (Carragher & Hancock, 2020). We included these example trials in the current experiment to give the participants some familiarity with the sAFRS before asking them to provide judgments about their trust in the system.

### Initial estimates of ability

After reading the task instructions, participants were asked to indicate their confidence in their face matching ability, estimate their accuracy on the task, and whether they believed their unassisted performance would be below or above that of the average human. We asked these three questions about individual ability before the baseline phase so that we could conduct exploratory analyses as to how perceived ability might change with task experience.

### Trust in automation questionnaires

Though we report them separately here, participants completed these components as a single questionnaire in the experiment. The questionnaire was administered twice, once after baseline but before the aided task phase (pre-assistance), and again after the aided task phase (post-assistance).

#### Relative trust in automation (Lee & Moray, 1994)

Our primary measure of relative trust in automation was derived from Lee and Moray’s (1994) two item questionnaire of trust in automation and self-confidence. Participants were asked to indicate their level of confidence in their ability to match faces accurately (self-confidence), along with their level of trust in the sAFRS to accurately judge whether two photographs show the same person (trust in automation). Both ratings were made on a scale from 0 to 10. Relative trust in automation was calculated by subtracting ratings of self-confidence from ratings of trust in the sAFRS.

#### Exploratory trust questionnaire items

We also asked a number of exploratory questions that were developed for the current project. These questions included some that were first asked in Experiment 1 (with minor wording changes to improve clarity), along with new questions for the current experiment, including a direct question about whether the participant trusted the AFRS to assist them in the task (yes, no).

### Procedure

Participants were told that the experiment was about humans using state-of-the-art facial recognition systems to perform face matching tasks. They started the experiment by completing the initial estimate of ability questions, before going on to complete the unassisted baseline phase of the face matching task. After completing the baseline task, the participants could take a short break, after which they were shown the sAFRS example trials, reminded that the sAFRS would give the correct answer on 95% of trials, and then asked to complete the pre-assistance trust in automation questionnaires. Participants then completed the aided face matching task with the assistance of the sAFRS. Finally, participants completed the post-assistance trust in automation questionnaires.

### Analyses

#### Face matching performance

Participants’ binary identification decisions (“same”, “different”) were used to calculate all possible measures of performance on the face matching task. Below, we analyse performance using overall accuracy, [((Hits + Correct Rejections)/Total Trials) * 100)].

#### Performance change

Several hypotheses specify that we will measure change in performance from baseline. However, it is important that we account for the fact that skilled individuals who perform highly at baseline will likely continue to do so in the aided test phase, such that they will have fewer trials on which they require the assistance of the sAFRS. As such, we calculated each participant’s change in performance when aided, as a percentage of improvement possible from baseline, [((*aided performance* minus *baseline performance*)/(*baseline errors*)) * 100]. Using this formula, an individual who correctly answers 26/40 trials at baseline and 33/40 trials when aided would have achieved 50% of the total improvement possible from baseline, as would an individual who correctly resolves 36/40 trials at baseline and 38/40 trials when aided. This measure was designed to account for each participant’s underlying face matching ability, while acknowledging that participants were shown different pairs of faces in the baseline and aided phases of the task. This measure does, however, mean that a handful of (typically highly skilled) individuals who experience a decrease in performance when aided by the sAFRS can record very large negative values on this measure (i.e. someone who answers 38/40 trials at baseline, but 36/40 when aided, will record a value of -100%).

#### Trust in automation

Relative trust in automation was measured using Lee and Moray’s (1994) two item trust/self-confidence measure. As in their original study, we subtracted self-confidence ratings from trust in automation ratings, to leave a change score in which positive values indicate higher trust in the automated system and negative values greater self-confidence. Therefore, this is a measure of relative trust in automation.

Additionally, several of our hypotheses were addressed by responses to questions developed for the current project. As in Carragher and Hancock (2023), we also asked participants questions related to trust in automation that were not the subject of planned analyses. All exploratory analyses are clearly identified in the results section.

Though participants completed the trust questionnaires twice (pre-assistance, post-assistance), we pre-registered using only the pre-assistance responses to test our hypotheses. The pre-assistance responses reflect the participant’s initial intentions towards the sAFRS, which have not yet been influenced by the experience of using it (Madhavan et al., 2006; Ross et al., 2008). Post-assistance responses were collected so that we could conduct exploratory analyses to examine changes in these ratings following sAFRS use.

### Data availability

The data analysed in Experiments 1 & 2 are available in our Open Science Framework (OSF) repository (https://osf.io/g3eqm/). The design, hypotheses, and analysis plan for Experiment 2 were pre-registered prior to data collection (https://osf.io/rjfup). The trust in automation questionnaire used in Experiment 2 is also available in the OSF repository.

We have deviated from our pre-registration by only reporting the results for overall accuracy in the main text, rather than also including the signal detection measures (Macmillan & Creelman, 2004; Stanislaw & Todorov, 1999) of d´ (sensitivity) and criterion (response bias). The rationale behind this change is simply that accuracy and sensitivity are very highly correlated in these data (Baseline: *r* = 0.97, *p* < 0.001; Aided: *r* = 0.97, *p* < 0.001), and the analysis of sensitivity leads to the same conclusions presented here. Nonetheless, these signal detection measures are included with the data files available online. Finally, while we have followed the analysis plan outlined in our pre-registration, we have used equivalent non-parametric tests where appropriate.

## Results

### Preliminary analyses

Initial examination of accuracy scores indicated that the data were negatively skewed in the baseline and aided task phases. While the distribution of accuracy scores at baseline was mesokurtic, the distribution of aided accuracy scores was leptokurtic. The change in aided performance as a percentage of improvement possible was also negatively skewed and leptokurtic. Given the non-normal distribution of these data, non-parametric analyses have been used where appropriate.

Average accuracy scores in both task phases were higher than those observed in Experiment 1 (see Table 1). Three participants demonstrated perfect accuracy during baseline (overall accuracy = 100%). Since we could not calculate a percentage of improvement possible score for these 3 individuals (requiring division by 0), they were omitted from the analyses for hypotheses H4–H7. For transparency, these 3 individuals achieved overall accuracy in the test phase of 92.5% (making 3 errors), 95% (2 errors), and 97.5% (1 error).

**Table 1:** Experiment 2 descriptive statistics

Variable	Mean	SD	Median	Min	Max
Baseline accuracy	82.2	9.5	85.0	52.5	100.0
Aided accuracy	91.3	6.9	92.5	60.0	100.0
Change in accuracy	9.1	9.3	7.5	− 12.5	32.5
Change in accuracy as % of improvement possible	42.7	55.8	55.6	− 300.0	100.0
Self-confidence	5.9	1.9	6.0	0.0	10.0
Trust in sAFRS	7.5	1.8	8.0	0.0	10.0
Relative trust in automation	1.56	2.3	1.0	− 5.0	8.0
Estimated unassisted accuracy	64.2	17.0	65.5	20.0	99.0
Estimated accuracy of sAFRS	88.0	14.9	95.0	19.0	98.0
Relative estimated accuracy	23.8	21.9	24.0	− 51.0	74.0
Predicted accuracy using sAFRS	81.5	14.5	85.0	22.0	100.0

### Planned analyses

#### H1

A Wilcoxon Signed Ranks test showed that participants improved their overall accuracy from baseline (*M* = 82.23, SD = 9.45) when assisted by the sAFRS at test (*M* = 91.30, SD = 6.91), *Z* = 9.11, *p* < 0.001. However, a one-sample Wilcoxon Signed Ranks test showed that aided performance failed to reach the level achieved by the sAFRS alone (95%), *Z* = − 6.29, *p* < 0.001. These results support H1 and replicate Carragher and Hancock (2023).

#### H2

**Fig. 1:**
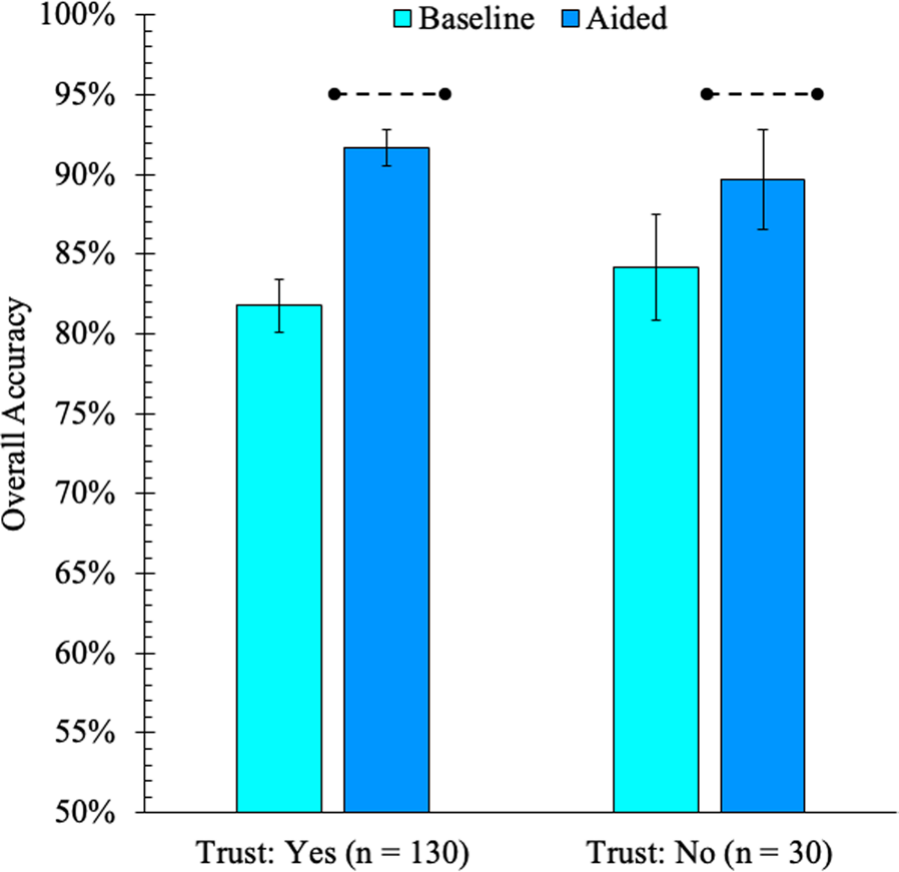
Overall accuracy in the baseline and aided task phases of Experiment 2, plotted separately by Trust Condition (yes, no). The accuracy of the sAFRS alone (95%) is indicated by the black dotted line above aided performance. Error bars show 95% confidence intervals

Participants were allocated to a Trust Condition based on their response to the pre-assistance question about whether they trusted the sAFRS (yes, no). In contrast to our expectations of an approximately even split, only 30 participants explicitly reported that they did not trust the sAFRS to help them in the task. A mixed ANOVA revealed a significant main effect of task phase, such that performance improved with assistance from the sAFRS (see H1), *F*(1, 158) = 68.07, *p* < 0.001, $\eta_{p}^{2}$ = 0.30. The main effect of trust condition was non-significant, *F*(1, 158) = 0.02, *p* = 0.894, $\eta_{p}^{2}$ = 0.00. Crucially, the interaction between the two factors was significant, *F*(1, 158) = 5.53, *p* = 0.020, $\eta_{p}^{2}$ = 0.03. As predicted, a Mann–Whitney test revealed there was greater improvement in accuracy when aided among participants who trusted the sAFRS (Median Improvement = 10.0), compared to those who did not (Median Improvement = 6.25), *Z* = 2.21, *p* = 0.027 (see Fig. 1). Please note, the difference in accuracy at baseline was not statistically significant, Z = 1.22, *p* = 0.222.

#### H3

Despite their greater improvement in performance, a one-sample Wilcoxon Signed Ranks test indicated that the aided performance of participants who trusted the sAFRS still failed to reach the level of performance achieved by the system alone (95%), *Z* = -3.29, *p* < 0.001. Likewise, the performance of participants who did not trust the sAFRS also failed to reach that of the system, *Z* = − 5.35, *p* < 0.001. Both results are consistent with our predictions.

#### H4

**Fig. 2:**
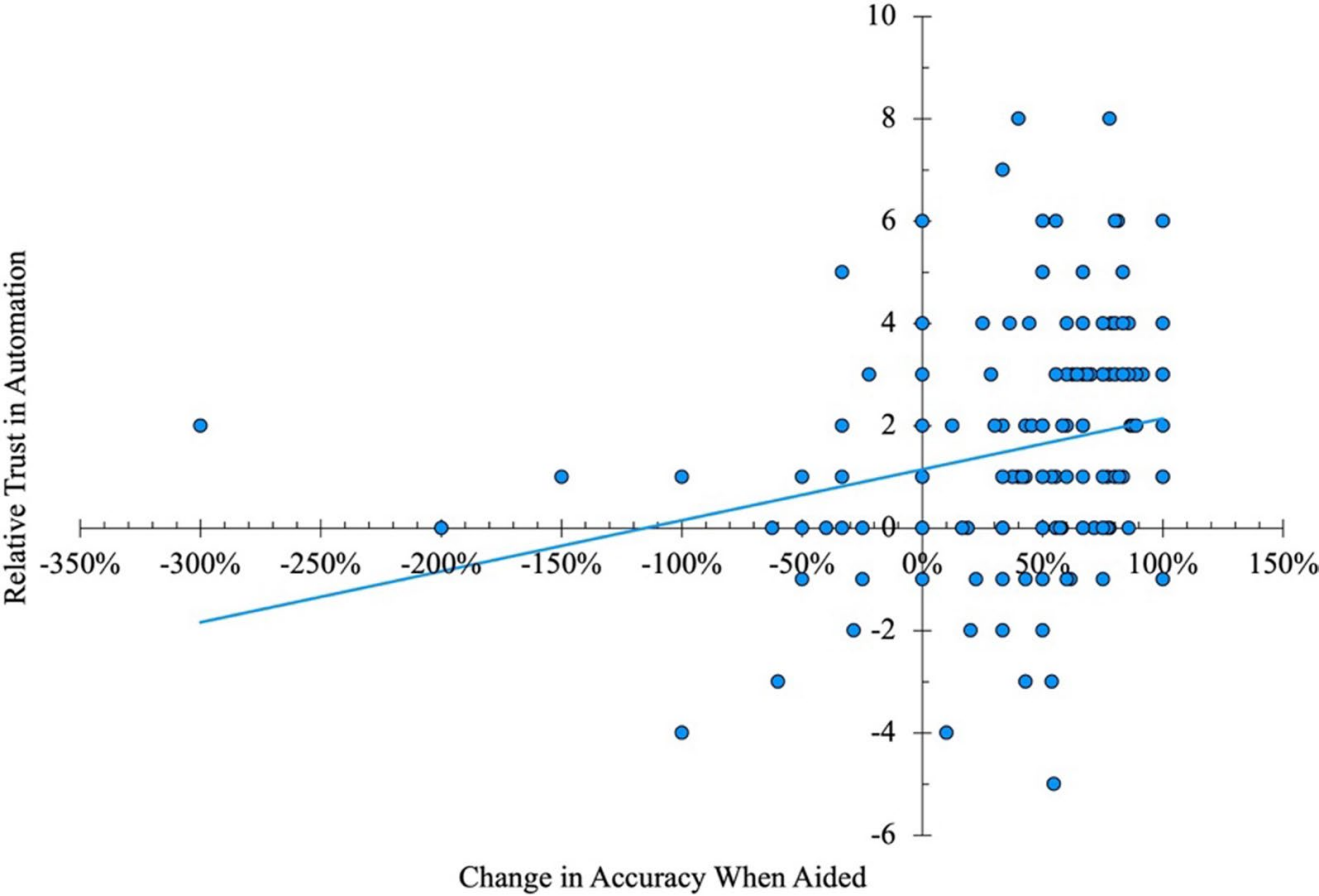
The relationship between change in accuracy when aided (as a percentage of improvement possible from baseline) and participants’ relative trust in automation (Trust in Automation minus Self-Confidence)

A relative trust in automation score (Lee & Moray, 1994) was calculated by subtracting participants’ self-confidence ratings from their trust in automation ratings. Positive relative trust scores represent greater trust in the sAFRS, while negative scores represent greater self-confidence. There was a significant moderate positive association between relative trust in automation and the change in accuracy when aided (as a percentage of improvement possible), *r*_*s*_ = 0.32, *p* < 0.001, supporting our prediction that greater relative trust in automation would be associated with greater improvements in performance when using the sAFRS as a decision aid (see Fig. 2).

#### H5

A relative estimated accuracy score was calculated by subtracting participants’ estimation of their own accuracy from their estimation of the sAFRS’s accuracy. Positive relative estimated accuracy scores signal that the sAFRS was estimated to be more accurate, while negative scores indicate that the participant estimated that they would be more accurate than the sAFRS. There was a significant weak-to-moderate positive association between relative estimated ability and performance change (as a percentage of improvement possible), *r*_*s*_ = 0.27, *p* < 0.001, supporting our hypothesis that perceptions of the sAFRS having greater accuracy would be associated with larger performance improvements when using the sAFRS as a decision aid (see Table 2).

**Table 2:** Spearman’s correlations for performance in Experiment 2

Variable	1	2	3	4	5	6	7	8	9
1. Baseline accuracy	–								
2. Aided accuracy	0.37**	−							
3. Change in accuracy	− 0.73**	0.29**	−						
4. Change in accuracy as % of improvement possible	− 0.24**	0.77**	0.77**	–					
5. Trust in sAFRS	0.08	0.25**	0.12	0.18*	–				
6. Self-Confidence	0.09	− 0.14	− 0.18*	− 0.17*	0.29**	–			
7. Relative trust in automation	− 0.06	0.29**	0.27**	0.32**	0.57**	− 0.56**	–		
8. Estimated unassisted accuracy	0.18*	0.01	− 0.17*	− 0.11	0.21**	0.64**	− 0.38**	–	
9. Estimated accuracy of sAFRS	0.09	0.28**	0.13	0.25**	0.61**	0.03	0.51**	0.17*	–
10. Relative estimated accuracy	− 0.15	0.16*	0.28**	0.27**	0.13	− 0.52**	0.61**	− 0.78**	0.35**

*p < 0.05, **p < 0.01

#### H6

Participants were allocated to a Perceived Superiority Condition (self, sAFRS) based on their response to a direct question about whether they or the sAFRS are better at face matching. Only 25 participants indicated that they would be superior to the sAFRS. Importantly, a Mann–Whitney test indicated that the perceived superiority conditions did not differ significantly in their baseline face matching performance, *Z* = 0.44, *p* = 0.661. As predicted, participants who expected the sAFRS to be superior improved more (as a percentage of improvement possible) when aided (Median Improvement = 60.0%) than those who thought that they would be superior (Median Improvement = 22.2%), *Z* = 3.41, *p* < 0.001.

#### H7

Participants were allocated to a Preferred Partner Condition (human, sAFRS) based on their response to a direct question about whether they would prefer a human or sAFRS partner if they were to do the task again. Only 33 participants indicated that they would prefer a human partner to the sAFRS. Importantly, the preferred partner conditions did not differ in their baseline face matching performance, *Z* = 0.05, *p* = 0.960. As expected, participants who would prefer to partner with an sAFRS improved more (as a percentage of improvement possible) when aided (Median Improvement = 58.3%), compared to those who preferred a human partner (Median Improvement = 40.0%), *Z* = 2.04, *p* = 0.042.

### Exploratory analyses

#### Self-confidence and performance

Participants were asked to indicate their confidence in their own face matching ability before the baseline phase (initial estimates), after the baseline phase (pre-assistance), and then again after the aided phase (post-assistance). Initial self-confidence was not significantly correlated with baseline accuracy (prospective), *r*_*s*_ = 0.12, *p* = 0.149. Similarly, pre-assistance confidence ratings were not significantly correlated with performance at baseline (retrospective), *r*_*s*_ = 0.09, *p* = 0.239, or with aided performance (prospective), *r*_*s*_ = -0.14, *p* = 0.088. Post-assistance confidence ratings were also not correlated with aided performance (retrospective), *r*_*s*_ = -0.09, *p* = 0.283. Taken together, these results suggest that general global confidence ratings are not well aligned with actual face matching ability.

#### Estimated accuracy and performance

At the same time points, participants also estimated their accuracy on the face matching task (0–100%). Initial estimates of accuracy were not significantly correlated with actual accuracy at baseline, *r*_*s*_ = 0.12, *p* = 0.115. There was, however, a weak positive correlation between predicted self-accuracy following the baseline phase and performance during the baseline phase, *r*_*s*_ = 0.18, *p* = 0.025. Similarly, there was a weak positive correlation between expectations of self-accuracy with assistance from the sAFRS prior to the test phase and test phase performance, *r*_*s*_ = 0.20, *p* = 0.011. These findings are consistent with the notion that participants have limited insight into their global face matching abilities (Bobak et al., 2019).

#### Estimated collaborative performance

Before starting the aided face matching task, participants estimated the level of accuracy they would achieve when assisted by the sAFRS. Curiously, despite being told that the sAFRS would give the correct decision on 95% of trials, the average prediction of aided accuracy was 81.5% (SD = 14.5). A total of 127 (79.4%) participants indicated that their aided accuracy would be lower than 95%, while a further 21 (13.1%) indicated that their aided performance would be exactly 95%. Only 12 (7.5%) participants predicted that their involvement in the task would improve the overall accuracy of the sAFRS. This pattern of results suggests that, on average, participants may have been aware that they would disregard correct decisions made by the sAFRS, despite knowing that this would result in lower performance than adopting a simple strategy of deferring all decisions to the sAFRS.

#### Change in perceived ability through the task

Participants initially estimated that they would achieve an unassisted accuracy of 71.94% (SD = 16.21). After completing the baseline phase of the experiment, estimates of individual accuracy dropped to 64.15% (SD = 16.97). Curiously, average performance at baseline was 82.23% (SD = 9.45), suggesting that participants overestimated the difficulty of the task. At the conclusion of the experiment, participants estimated that their accuracy in the aided phase *without the assistance of the AFRS* would have been 69.08% (SD = 17.01). Future research is needed to investigate whether this apparent increase in perceived ability represents a return to baseline, or whether it is due to the experience of using the sAFRS.

Participants’ confidence in their own face matching ability was higher after completing the task with assistance from the sAFRS (*M* = 6.26, *SD* = 2.02) than it was after baseline (*M* = 5.90, SD = 1.89). Likewise, participants’ trust in the sAFRS was also higher after using the sAFRS (*M* = 7.78, SD = 1.72) than it was before the aided phase (*M* = 7.46, SD = 1.83). These results suggest that interacting with the sAFRS can influence perceptions of ability, both of the self and the automated system.

#### Change in attitudes following sAFRS assistance

Before the aided phase, 30 participants indicated that they did not trust the sAFRS to help them with the task. Following the aided phase, 14 of these participants (46.7%) responded that they trusted the sAFRS had helped them. In contrast, of the 130 participants that initially trusted the sAFRS to help them, only 6 (4.6%) later reported that the sAFRS had not helped them during the task.

Of the 134 participants who initially believed the sAFRS would be more accurate than them, only 19 (14.2%) indicated that they were more accurate than the sAFRS following the task. In contrast, 9 (34.6%) of the 26 participants who initially believed that they would be more accurate changed their response after the aided task phase.

Of the 34 participants who initially indicated a preference for a human partner, 11 (32.4%) changed their preference to be partnered with a sAFRS following the aided task phase. In contrast, only 3 (2.4%) of the 126 participants with an initial preference for a sAFRS partner changed their preference to a human partner following the aided phase.

#### Correcting sAFRS errors

Participants saw the sAFRS make two errors during the aided task phase (one match trial, one mismatch trial). Here we investigate whether trust in the sAFRS might have led to greater acceptance of these errors. Importantly, although participants only saw the sAFRS make two errors during the aided phase, the sAFRS was programmed to make four errors across the entire GFMT2-S (due to counterbalancing). Below we compare accuracy on the two error trials that were shown at baseline (i.e. without the erroneous decision from the sAFRS) and the two error trials shown during the aided task phase with the incorrect decision from the sAFRS.

At baseline, participants who trusted the sAFRS (*n* = 130) answered an average of 1.55 (SD = 0.58) of the 2 error trials correctly, while participants who did not trust the sAFRS (*n* = 30) correctly answered 1.57 trials (SD = 0.57), *Z* = 0.05, *p* = 0.959. However, during the aided phase of the experiment, participants who trusted the sAFRS only correctly answered an average of 1.06 (SD = 0.67) error trials out of 2, whereas those who did not trust the sAFRS correctly answered 1.33 (SD = 0.66), *Z* = 2.02, *p* = 0.044. This pattern of results provides preliminary evidence that on average, participants were less accurate when trials were shown with an incorrect decision from the sAFRS, but that this decline was particularly evident among participants who reported trusting the sAFRS compared to those who did not.

#### Individual differences in aided accuracy

Although the average aided performance of participants failed to exceed the performance of the sAFRS alone (95%), there was a subset of participants (*n* = 74) who managed to achieve or exceed this level of performance when aided by the system. Curiously, the participants who achieved aided accuracy of exactly 95% (*n* = 37) did not simply accept the decision of the sAFRS on every trial (although some did, *n* = 8); among this group, some participants (*n* = 8) actually corrected both errors from the sAFRS, but then overturned two correct sAFRS decisions. A further 22 participants achieved aided accuracy of 97.5%, while 15 participants (9.4% of the total sample) achieved 100% accuracy when aided by the sAFRS.

When attempting to characterise the attributes of these high performing individuals, we can see that the baseline accuracy of the participants who achieved aided accuracy of 95% or more was greater (*M* = 85.7, SD = 7.6) than those who failed to achieve aided performance equal or exceeding that of the sAFRS (*M* = 79.2, SD = 9.9). However, baseline accuracy did not appear to differ between those who achieved aided accuracy of exactly 95% (*M* = 85.1, SD = 8.3), 97.5% (*M* = 86.4, SD = 6.7) or 100% (*M* = 86.2, SD = 7.3). When considered alongside the general finding that trust in automation (*r*_*s*_ = 0.25, *p* = 0.001) and relative trust in automation (*r*_*s*_ = 0.29, *p* < 0.001) were both positively correlated with accuracy in the aided task phase, these results suggest that individuals who show the greatest levels of performance with sAFRS assistance are those who are already quite skilled at face matching and have high levels of trust in automation.

## Discussion

All seven of our pre-registered hypotheses were supported. The average participant made significant accuracy gains when using the sAFRS as a decision aid, replicating Carragher and Hancock (2023). Further, those participants who explicitly reported trusting the sAFRS improved more with the assistance of the sAFRS compared to those who did not. Similarly, participants with greater relative trust in automation improved more with sAFRS assistance than those with lower trust in automation or greater self-confidence. Despite the benefit of trusting the sAFRS, the average assisted performance of both trust conditions failed to reach the level of accuracy achieved by the sAFRS alone. This pattern of results is indicative of sub-optimal collaborative decision-making (Bartlett & McCarley, 2017; Boskemper et al., 2021), arising due to failures correcting errors from the sAFRS or overturning some of the system’s correct decisions. Indeed, our exploratory analyses suggested that participants who trusted the sAFRS were more likely to endorse the system’s erroneous decisions compared to those with less trust in the system, a potential example of automation misuse (Parasuraman & Riley, 1997). This result provides interesting nuance to Howard et al.’s (2020) finding that participants were biased towards confirming algorithm decisions, regardless of the accuracy of the output. Nonetheless, nearly half (46.3%) of our participants achieved assisted performance equal to, or exceeding, the sAFRS’s accuracy of 95%, with 15 participants achieving 100% accuracy on the aided task. This finding suggests that a subset of individuals may employ highly efficient collaborative decision-making strategies when using the sAFRS as a decision aid.

## General discussion

Across two experiments, we investigated whether trust in automation was related to the level of performance participants achieved when using a sAFRS as a decision aid in a face matching task. Using a range of measures that were both established, such as relative trust in automation (Lee & Moray, 1994), and novel, including questions about perceived superiority (self or sAFRS) and preferred face matching partner (human or sAFRS), our results consistently showed that participants who gave responses that were more favourable towards the sAFRS achieved greater performance when using it as a decision aid. Our findings suggest that much like face matching ability (e.g. Bobak et al., 2016b; Burton et al., 2010), there are individual differences in the ability to effectively use AFRS as decision aids.

Most participants experienced significant improvements to their overall accuracy when using the sAFRS as a decision aid. These findings are consistent with previous research showing that human decisions are biased towards those of an AFRS (Barragan et al., 2022; Fysh & Bindemann, 2018a; Howard et al., 2020). When the AFRS has higher accuracy than most human participants, this tendency will generally lead to improved human performance (Carragher & Hancock, 2023). The average participant, however, failed to achieve a collaborative performance gain when assisted by the sAFRS. Specifically, human intervention was likely to result in lower performance than if the sAFRS had been left to complete the task alone. Both results replicate our previous findings (Carragher & Hancock, 2023), in a new face matching task, speaking to the generalisability of these effects. The current study shows that even among individuals with favourable attitudes towards the sAFRS, aided performance is not certain to reach, let alone exceed, the capabilities of the system alone. While our results show that a sAFRS with accuracy higher than the average human is an effective decision aid that can improve human performance, these findings raise further questions about the efficacy of human–algorithm teams for face identification (White et al., 2015a).

The vast majority of participants (*n* = 130) indicated that they trusted the sAFRS to help them with the face matching task, when asked directly in the pre-assistance questionnaire. While participants who trusted the sAFRS experienced greater improvement in performance than those who did not (*n* = 30), this improvement in personal performance potentially came at a cost to collaborative task performance. Our exploratory analyses suggested that these trusting participants were more likely to endorse incorrect decisions from the sAFRS. These results demonstrate the difficulty of evaluating the efficacy of human–algorithm teaming in this context. That is, when the decision aid is considerably more accurate than the average human, most participants could significantly improve their own performance simply by confirming every decision made by the sAFRS, including those that are errors. However, this strategy would make the role of the human in this workflow redundant, as they would fail to overturn any errors from the sAFRS. As we have previously noted (Carragher & Hancock, 2023), optimal human–algorithm teaming will result in a level of collaborative performance exceeding that which the algorithm achieves alone. Our results suggest that in scenarios where the decision aid is often correct, high trust in automation—which is important if the human is to accept the many correct decisions from the sAFRS—can resemble the complete, uncritical, dependence on the decision aid, which renders human involvement pointless. Therefore, it is important to remember that the efficacy of human–algorithm teams should not be measured by the improvement experienced by the human, but by whether collaborative performance exceeds that offered by either agent alone.

Nonetheless, we did find a subset of participants who achieved levels of aided performance that were equal to, or exceeded, that of the sAFRS alone. Our preliminary investigation suggested that these participants were relatively skilled at face matching, as indicated by their high accuracy at baseline, and had high levels of trust, and relative trust, in automation. These attributes are not surprising when we consider the nature of the aided task. A skilled individual with low trust in the sAFRS may disuse the system by overturning its correct responses, whereas a less skilled individual who is trusting of the sAFRS may misuse the system by failing to correct erroneous decisions (Parasuraman & Riley, 1997). Optimal collaborative performance can only be achieved if the observer accepts the system’s correct decisions and overturns its incorrect decisions (Bahrami et al., 2010), a level of calibration likely to require both individual skill and trust in the aid. Researchers have previously suggested that professional face matching roles (e.g. border control) would be well served by recruiting individuals known to be skilled at face matching (Bobak et al., 2016a). Similarly, our findings suggest that there may be individuals who are particularly well suited to professional identification roles that require human–algorithm teaming. While our data suggest that these observers are skilled face matchers who are also trusting of automation, additional research is needed to further characterise the attributes of these individuals, so that they can assessed for suitability in operational contexts.

When measured pre-assistance, trust in the sAFRS and self-confidence were both associated with face matching performance when using the sAFRS as a decision aid. But our exploratory analyses suggested that trust in the sAFRS and self-confidence also increased after using the sAFRS. As such, experience using the sAFRS might influence operators’ perceptions, both of the system and their own ability. This suggestion is further highlighted by the fact that following the aided task phase, 46.7% of participants who initially did not trust the sAFRS reported that the sAFRS had helped them during the task. These findings are consistent with Hoff and Bashir’s (2015) concepts of situational and learned trust. Situational trust is influenced by external variables, such as task difficulty and the perceived benefits of automation (Madhavan et al., 2006; Ross et al., 2008), as well as internal factors such as self-confidence (Lee & See, 2004). Learned trust is then developed through exposure to the automated system (Hoff & Bashir, 2015). Together, our findings suggest that relative trust in the sAFRS is not only dependent on the human’s initial perceptions of the system, but also on the human’s experience of using the system. As such, future research might examine the algorithm-assisted face matching performance of participants with previous experience using such systems.

Participants were told several times during the experiment that the sAFRS would show the correct identification decision on 95% of trials (in fact, the analyses consisted only of participants who correctly answered an attention check question about the stated accuracy of the sAFRS). Yet, the average aided performance of participants failed to reach this level of performance, replicating our previous findings (Carragher & Hancock, 2023). These data are potentially consistent with the common cognitive bias of probability neglect (Rottenstreich & Hsee, 2001; Sunstein, 2002). That is, participants may have disregarded the information that the sAFRS was 95% accurate when estimating the assistance that the system would provide on each trial. Interestingly, our data also suggest that the participants may have been aware that they were going to overturn some correct decisions from the sAFRS. When asked how accurate they would be when assisted by the sAFRS, the average estimate was just 81.5%. Moreover, only 7.5% of participants expected that partnering with the sAFRS would produce a collaborative performance gain, while 79.4% of the sample expected that they would limit the accuracy of the system. These assisted accuracy estimates were positively correlated with actual assisted accuracy. Further research is needed to investigate whether participants intentionally reported that they would limit the performance of the sAFRS, and if so, to uncover why such a belief existed among novice participants.

Interestingly, the proportion of participants reporting that they would rather complete the task again with the assistance of the sAFRS (as opposed to a human) differed between Experiment 1 (49.5%) and Experiment 2 (78.7%). It is not immediately obvious why the two samples differed so considerably, particularly when they were recruited from the same online platform. One possibility is that by including the example sAFRS trial displays in the instructions for Experiment 2, participants were able to see the types of difficult face matching trials that the sAFRS can resolve correctly, potentially leading to an increase in their perception of its ability. Alternatively, a speculative suggestion is that attitudes towards sAFRS (or perhaps Artificial Intelligence more generally), may have changed between the periods of data collection for the two projects (Experiment 1: Q3 2021; Experiment 2: Q1 2023). Whatever the reason for this discrepancy, there were fewer participants who were openly distrusting of the AFRS in Experiment 2, leading to a smaller than expected sample size for the “no” trust condition. Nonetheless, as our participants were members of the general public, this sample should reflect the beliefs held among the wider population around AFRS, who may one day find themselves in professional roles requiring algorithm-assisted identification as these technologies are introduced into different settings and workplaces (Noyes & Hill, 2021; Ritchie et al., 2021).

One curiosity in these data relates to the average performance on the GFMT2-S in Experiment 2. As expected, baseline accuracy did not differ between Set A (*M* = 81.6, SD = 9.26) and Set B (*M* = 82.9, SD = 9.65), *p* = 0.361. However, average accuracy appears higher than reported by White et al. (2022) when creating the GFMT2-S (Set A: *M* = 76.4, SD = 9.1; Set B: *M* = 76.4, SD = 7.9). The reason for this discrepancy is not obviously apparent. However, the difference does mean that the face matching task was easier than intended, and easier than the EFCT used by Carragher and Hancock (2023). As such, it is possible that the large number of participants who achieved high levels of aided performance may have been due to face matching task being easier than expected. Future research is needed to determine whether these high levels of aided performance persist on harder face matching tasks. Nevertheless, it is notable that even with an easier face matching task, the aided performance of the average participant still did not exceed the level of accuracy achieved by the AFRS alone.

A limitation to this study is that our participants were novices, recruited online from the general population (as was the case in Carragher & Hancock, 2023). While working in a profession that regularly performs face matching does not automatically lead to improved face matching abilities (White et al., 2014; c.f. Wirth & Carbon, 2017), there are certain face identification professionals—“forensic facial examiners”—who do show exceptional performance (Phillips et al., 2018; White et al., 2015b). We found that the participants who were able to achieve high levels of performance when assisted by the sAFRS tended to have high face matching accuracy at baseline. One possibility arising from our research is that some professionals—those with exceptional face matching abilities—might be better suited to roles that involve use or oversight of AFRS than others. Moreover, we found that trust in automation increased after participants completed the face matching task with assistance from the sAFRS. This finding raises the possibility that professionals who regularly interact with AFRS might show higher levels of trust in automation than were seen among our sample of participants. While both suggestions are speculative, they both demonstrate the need for future research to investigate the algorithm-assisted face matching performance of professionals who have experience using facial recognition technologies. Nonetheless, the current results still speak to the potential difficulties of recruiting individuals to professional roles that include AFRS use, or in introducing AFRS into tasks that were previously completed by humans.

A second limitation is our use of a simulated AFRS in these experiments. As noted in the introduction, we describe the AFRS as being “simulated” because we introduced additional errors into its performance that the real DCNN facial recognition algorithm did not make. We gave the sAFRS accuracy of 95% on the GFMT2-S, which is far higher than that of the average human (White et al., 2022). While this performance appears to be considerably lower than the > 99.9% accuracy recorded by the best algorithms systems in current 1:1 verification testing with high quality imagery (National Institute of Standards and Technology, 2024), these numbers may not be directly comparable, since many algorithm tests generate false matches by comparing every face to every other in a database, whereas the pairs in face matching tasks for humans are deliberately chosen to be difficult. Nonetheless, real state-of-the-art AFRS may be more accurate than the sAFRS used here. Moreover, we have previously seen that human and AFRS performance is correlated, such that both are more likely to make mistakes on the same face pairs (Carragher & Hancock, 2023). Taken together, these points mean that although our current sample revealed a substantial proportion of participants who achieved or exceeded the level of performance of the sAFRS alone when assisted by the system, they did so under relatively favourable conditions. Further research using a state-of-the-art AFRS, and a highly challenging face matching task, is needed to examine just how many participants are likely to achieve a collaborative performance gain under realistic task conditions.

## Conclusions

Very few studies have investigated human–algorithm teaming in the context of one-to-one face matching tasks. While previous research has shown that human decisions are biased towards those of the AFRS (Barragan et al., 2022; Fysh & Bindemann, 2018a; Howard et al., 2020), which can lead to improved participant performance when the aid is more accurate than most humans (Carragher & Hancock, 2023), we believe that this is the first study to examine individual differences in the algorithm-assisted face matching performance of participants. Across two experiments, we show that individuals with greater trust in automation experience greater improvements in their face matching performance when assisted by the sAFRS compared to participants with lower trust in automation (or greater self-confidence). However, the aided performance of the average participant failed to reach the level of performance offered by the sAFRS alone, regardless of their trust in automation condition. This result is consistent with our previous research (Carragher & Hancock, 2023), and offers further support for the suggestion that the human can be a factor limiting the performance of the AFRS (White et al., 2015a). Nonetheless, we identified a relatively skilled subgroup of participants who were able to achieve collaborative accuracy gains when working with the sAFRS. Though additional research is required to test the performance of these participants when assisted by state-of-the-art AFRS on even more challenging face matching tasks, the emergence of this subgroup offers hope that there may be routes to effective models of human–algorithm teaming in the context of face identification.

## Data Availability

The data sets generated and analysed in the current study are available in the OSF repository [https://osf.io/g3eqm/].

## Abbreviations

AFRS: Automated facial recognition system
DCNN: Deep convolutional neural network
sAFRS: Simulated automated facial recognition system
EFCT: Expertise in facial comparison test
GFMT2-S: Glasgow face matching test 2, short version
